# Clinical application of synovial fluid albumin-to-globulin ratio as a novel biomarker in diagnosing chronic periprosthetic joint infection

**DOI:** 10.3389/fcimb.2026.1750599

**Published:** 2026-05-22

**Authors:** Qianshui Hu, Lin Yang, Rui Zhang, Yaji Yang, Haotian Zhou, Nianshuang Wei, Haiqing Chen, Fabo Zhu, Dijiao Tang, Leilei Qin, Ning Hu

**Affiliations:** 1 Department of Orthopaedic Surgery, The First Affiliated Hospital of Chongqing Medical University, Chongqing, China; 2 Chongqing Municipal Health Commission Key Laboratory of Musculoskeletal Regeneration and Translational Medicine, Chongqing, China; 3 Orthopaedic Research Laboratory, Chongqing Medical University, Chongqing, China; 4 Chongqing Medical University, Chongqing, China; 5 Department of Laboratory Medicine, The First Affiliated Hospital of Chongqing Medical University, Chongqing, China

**Keywords:** albumin-to-globulin ratio, diagnosis, periprosthetic joint infection, revision arthroplasty, synovial fluid biomarkers

## Abstract

**Background:**

Periprosthetic joint infection (PJI) is one of the most common and severe complications following joint arthroplasty. Early diagnosis is essential for timely and effective clinical intervention and carries great clinical significance. This study aims to evaluate the value of the albumin-to-globulin ratio (AGR) in synovial fluid (SF) for diagnosing PJI and compare its diagnostic performance with traditional biomarkers such as serum C-reactive protein (CRP), erythrocyte sedimentation rate (ESR), synovial fluid polymorphonuclear percentage (SF-PMN%), and the potential marker serum albumin-to-globulin ratio (SE-AGR).

**Methods:**

This prospective study enrolled 230 patients who experienced pain following total hip or total knee arthroplasty, including 97 cases of PJI and 133 cases of aseptic failure(AF). Blood samples and synovial fluid aspirates were collected within 24 hours preoperatively. The association between each variable and PJI was evaluated using univariate logistic regression analysis, and the diagnostic performance of each indicator was assessed through receiver operating characteristic (ROC) curve analysis.

**Results:**

The results of the study showed that the Wald χ^2^ value of the synovial fluid albumin-to-globulin ratio (SF-AGR) was 47.1, which was higher than that of CRP (43.7) but lower than those of SE-AGR (54.0), ESR (56.5), and SF-PMN% (49.0). Similar to other conventional biomarkers, SF-AGR demonstrated a significant association with PJI. The area under the curve (AUC) of SF-AGR was 0.9504, which was superior to SE-AGR (AUC = 0.8682), ESR (AUC = 0.8427), CRP (AUC = 0.8507), and SF-PMN% (AUC = 0.8843), indicating that SF-AGR showed the most outstanding performance in the preoperative diagnosis of PJI.

**Conclusion:**

SF-AGR shows promise as a novel biomarker for the diagnosis of PJI, supporting its potential use as an adjunct to current diagnostic approaches. Diagnosis, Periprosthetic Joint Infection, Albumin-to-Globulin Ratio, Synovial Fluid Biomarkers, Revision arthroplasty.

## 1 Introduction

Prosthetic Joint Infection (PJI) is among the most serious complications following joint arthroplasty, with an incidence ranging from 0.88% to 2.18% (Tande and Patel, 2014; Marang-van de Mheen et al., 2017; Boyle et al., 2020). PJI leads to severe pain and functional disability, and in more severe cases, it can result in systemic infections that may require joint excision or amputation. This condition imposes a significant burden on both the patient’s quality of life and the associated healthcare costs (Kurtz et al., 2012; Zmistowski et al., 2013). Pathogens, due to their immune evasion mechanisms and resistance mutations, often lead to the development of chronic PJI with subtle clinical manifestations, making it difficult to distinguish affected patients from those with aseptic failure (Qin et al., 2024). These subtle clinical manifestations can result in delays in the recognition of chronic PJI. Accurate preoperative diagnosis of chronic PJI is therefore essential for guiding surgical planning, including the extent of intraoperative irrigation and debridement, and for determining the appropriate use of intraoperative and postoperative broad-spectrum antibiotics rather than prophylactic antibiotics alone. Although the diagnostic criteria for PJI are updated annually by various global societies, a gold standard for diagnosis is still lacking. This may be related to immune evasion and biofilm formation by infecting organisms, which can lead to relatively mild or nonspecific clinical manifestations and make differentiation from aseptic failure more challenging (Costerton et al., 1999; Uhel et al., 2019; Qin et al., 2024). Furthermore, aseptic loosening accompanied by metal particle release induces local aseptic inflammation in the joint, making it difficult to differentiate from PJI based on clinical symptoms (Hallab and Jacobs, 2009). C-reactive protein (CRP) and erythrocyte sedimentation rate (ESR) are widely used as systemic inflammatory markers to reflect the body’s inflammatory response and have been incorporated into diagnostic consensus for PJI, such as the 2013 MSIS and 2018 ICM guidelines (Parvizi et al., 2018; Villa et al., 2020). However, these serum biomarkers are susceptible to systemic interference and lack specificity. Moreover, their diagnostic value is lower in patients with culture-negative PJI compared to those with culture-positive PJI (Malekzadeh et al., 2010; Watanabe et al., 2021; Su et al., 2022). Although microbial culture is considered the definitive diagnostic standard for PJI, its accuracy is often influenced by sample collection methods and timing. Additionally, prior antibiotic use in some patients can lead to false-negative culture results (Palan et al., 2019; Li et al., 2020). Although imaging modalities such as X-ray, CT, and PET-CT are also employed in the diagnosis of PJI, they are limited by issues including metal artifacts, high costs, complex procedures, and suboptimal sensitivity and specificity, and therefore do not fully address the challenges in PJI diagnosis (Doweiko and Nompleggi, 1991; Hoveidaei et al., 2024; Gu et al., 2025). Therefore, clinicians urgently need to identify new diagnostic markers that offer high diagnostic performance while remaining cost-effective for PJI diagnosis.

Recently, the serum albumin-to-globulin ratio (SE-AGR) has attracted considerable attention in the literature due to its low cost, rapidity, and minimally invasive nature in the diagnosis of PJI. Albumin and globulin are among the major protein components in the human body. Albumin is primarily synthesized by the liver and functions to maintain plasma oncotic pressure, transport various substances, and modulate immune responses (Wang et al., 2021). Globulins include multiple immunoglobulin classes and play a central role in immune defense (Gabay and Kushner, 1999). As the ratio of albumin to globulin, AGR reflects the overall immune and inflammatory status of the body, with lower values typically associated with impaired immune function and heightened inflammatory activity (Dong et al., 2024). Previous studies have shown that serum AGR exhibits good sensitivity for diagnosing joint infections; however, compared with conventional biomarkers such as CRP and ESR, its specificity is relatively limited, likely due to interference from systemic inflammation, nutritional status, and other infectious conditions (Hui et al., 2012). Similar to other serum biomarkers, AGR lacks specificity for joint-specific infections.

Synovial fluid, as a specimen from within the joint, can directly reflect the inflammatory process in the joint and is less influenced by systemic inflammation (Qin et al., 2020).Studies have demonstrated that synovial fluid biomarkers are more sensitive for diagnosing joint infections. In our previous research, we also found that synovial fluid IL-6 has higher specificity than serum IL-6 in diagnosing PJI (Zhang et al., 2021). Although the potential of serum AGR has been demonstrated in diagnosing pyogenic liver abscesses, pyogenic spondylitis, and infectious tibial nonunion, no studies have specifically explored the role of AGR in synovial fluid for diagnosing PJI (Boyle et al., 2018; Wang et al., 2023; Mitsui et al., 2025). Therefore, we pose the question of whether the AGR in synovial fluid holds promise for diagnosing PJI. By analyzing the AGR levels in synovial fluid, this study aims to (Tande and Patel, 2014): determine the optimal cutoff value of synovial fluid albumin-to-globulin ratio (SF-AGR) for diagnosing PJI; and (Boyle et al., 2020) evaluate the diagnostic performance of SF-AGR compared to other diagnostic standards, including serum AGR, ESR, CRP, and synovial fluid polymorphonuclear percentage (SF-PMN%). This research indicates that SF-AGR shows promise in improving the accuracy of PJI diagnosis and may provide additional support for clinical decision-making, but these results require further validation in future studies.

## 2 Materials and methods

### 2.1 Patient cohort and characteristics

**Figure 1:**
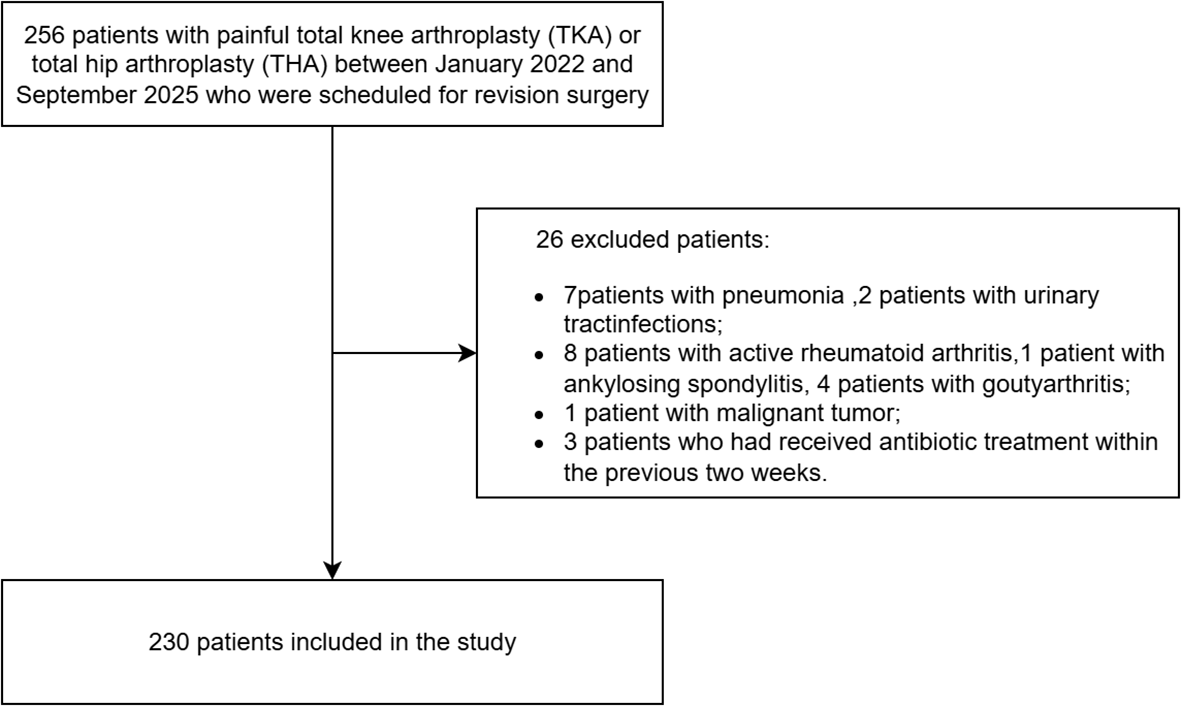
Flowchart of patient selection from the local database.

This study is a prospective investigation that has been approved by the Institutional Ethics Committee of the First Affiliated Hospital of Chongqing Medical University (20187101), and all patients have signed informed consent forms. We included 256 patients who experienced pain occurring 6 weeks after undergoing total knee arthroplasty (TKA) or total hip arthroplasty (THA) between January 2022 and September 2025 and were scheduled for revision surgery. According to the 2013 Musculoskeletal Infection Society (2013 MSIS) criteria, the patients were divided into the PJI group and the aseptic revision group. PJI was considered chronic PJI when PJI symptoms occurring 6 weeks after surgery. The aseptic failure group was defined as cases undergoing revision due to non-infectious causes, including aseptic loosening, wear, instability, dislocation, adverse local tissue reactions, and metal hypersensitivity reactions. To eliminate potential confounding factors that might affect diagnostic accuracy, a total of 26 patients were excluded from the study: (1) patients with other organ infections, including pneumonia (n = 7) and urinary tract infections (n = 2);(2)patients with active rheumatoid arthritis (n = 8), ankylosing spondylitis (n = 1), or gouty arthritis (n = 4); (3) patients with malignant tumors (n = 1); and (4) patients who had received antibiotic treatment within two weeks prior to sampling (n = 3). Patients who underwent synovial fluid aspiration but yielded no sample (‘dry tap’) were not included in the analysis, as measurement of synovial biomarkers was not possible. Only patients with sufficient synovial fluid for biomarker assessment were included in the study. Ultimately, 230 patients were included in the final analysis (**Figure 1**).

Upon admission, baseline data for all patients were recorded, including age, weight, height, and sex. Before the revision surgery, peripheral venous blood samples (3 mL) were collected from all patients to measure ESR,CRP levels, and SE-AGR. Additionally, synovial fluid samples (3–4 mL) were collected for biochemical analysis, with a focus on determining the AGR and the percentage of polymorphonuclear neutrophils. Microbial cultures were performed on preoperative synovial fluid samples and at least three tissue samples collected from different surgical sites, using standard culture (48-hour incubation) and extended culture (14-day incubation) methods. All cultures were conducted under standard microbiological laboratory conditions.

### 2.2 Sample measurement

Fasting venous blood samples were routinely collected by nurses on the second day after admission to measure relevant serum biomarkers. All blood samples were delivered to the hospital laboratory within one hour after collection for preoperative analyses. Synovial fluid samples were obtained prior to revision arthroplasty and were required to be processed and frozen within one hour to prevent proteolytic degradation. All synovial fluid specimens were collected into sterile syringes and centrifuged within one hour to remove cellular and particulate components. The resulting cell-free supernatants were immediately frozen on dry ice and stored at −80 °C until final analysis.

All biochemical measurements were performed in the hospital’s Biotechnology Platform Laboratory. The tested indicators included SF-AGR, SE-AGR, CRP, ESR, and SF-PMN% in both serum and synovial fluid to evaluate their diagnostic potential for PJI. Specifically: SF-AGR and SE-AGR were measured using an automated biochemistry analyzer, Roche Cobas 8000 c702 (Roche Diagnostics, Basel, Switzerland) with standard albumin and globulin reagents. CRP was determined by a particle-enhanced turbidimetric immunoassay using a HITACHI 7600 Series Automatic Biochemical Analyzer (Hitachi, Tokyo, Japan) and diagnostic kits from DiaSys Diagnostic Systems GmbH (Shanghai, China). SF-PMN% was assessed using an automated hematology analyzer, Sysmex XN-1000 (Sysmex Corporation, Kobe, Japan) following centrifugation to remove cellular debris. ESR was measured using an automated erythrocyte sedimentation rate analyzer, BP200n (Alifax, Padua, Italy).

### 2.3 Data analyses

Statistical analysis was performed using SPSS version 25 software (IBM Corp., Armonk, NY, USA) and GraphPad Prism 9.0 software (GraphPad Software, San Diego, CA, USA). Categorical variables are presented as counts and percentages, and statistical significance was assessed using the chi-square test. Continuous variables with a normal distribution are expressed as mean ± standard deviation, and statistical significance between two groups was determined using the parametric t-test. For non-normally distributed variables, the interquartile range (IQR) was used to describe the data, and the Mann-Whitney U test was employed for comparisons. Univariate logistic regression analyses were performed to evaluate the association between each biomarker and the presence of PJI. Odds ratios (ORs) and 95% confidence intervals (CIs) were calculated. Receiver operating characteristic (ROC) curves were plotted using GraphPad Prism 9.0, and the area under the curve (AUC), sensitivity, specificity, positive predictive value, negative predictive value, and accuracy were calculated to evaluate the diagnostic value of biomarkers for PJI. The Youden J statistic was used to determine the optimal threshold for biomarkers distinguishing PJI from aseptic loosening (J = sensitivity + specificity - 1). A p-value of <0.05 was considered statistically significant.

## 3 Result

### 3.1 Baseline characteristics of patients

**Table 1** shows the demographic characteristics of the two patient groups. A total of 230 patients were included, with 133 patients in the aseptic failure (AF) group and 97 patients in the PJI group. No statistically significant differences were observed between the two groups in terms of baseline characteristics, including age (P = 0.2090), weight (P = 0.3015), height (P = 0.2811), and BMI (P = 0.8853). Regarding gender distribution, no significant differences were found between the PJI and aseptic loosening groups in the proportion of males and females (P = 0.7867).Similarly, no significant differences were observed in joint type distribution, with comparable proportions of knee and hip joints between the two groups (P = 0.2658).

**Table 1:** The demographic data of the study population.

Characteristic		AF (n=133)	PJI (n=97)		P value
Age(years)		65.76 ± 9.76	64.20 ± 8.61		0.2090
Weight(kg)		62.65 ± 9.08	63.95 ± 9.74		0.3015
Height(m)		1.61 ± 0.08	1.62 ± 0.07		0.2811
BMI(kg/m^2^)		24.34 ± 4.20	24.26 ± 4.14		0.8853
Sex					0.7867
Man	57	42.86%	39	40.21%	
Woman	76	57.14%	58	59.79%	
Join Type					0.2658
Knee	82	61.65%	67	69.07%	
Hip	51	38.35%	30	30.93%	

Variables are expressed as mean ± SD, or numbers (percentage).BMI, body mass index. PJI, periprosthetic joint infections; AF, aseptic failure. Statistically significant. (P-value ≤ 0.05).

### 3.2 Pathogen distribution in PJI patients

**Figure 2:**
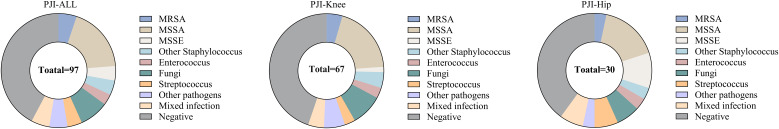
Pathogen detection results. MSSA, methicillin-susceptible Staphylococcus aureus; MRSA, methicillin-resistant Staphylococcus aureus; MSSE, methicillin-susceptible Staphylococcus epidermidis; Enterococcus, enterococci; Fungi, fungal pathogens; Streptococcus: Streptococcus spp.; Other pathogens, other bacterial species; Mixed infection, multiple pathogens in the same patient; Negative, culture-negative PJI cases.

As shown in **Figure 2**, the overall and joint-specific distributions of pathogens in PJI patients are illustrated. Among the 97 patients with PJI, 56 (57.7%) were culture-positive, while 41 (42.3%) were culture-negative. In the total cohort, the most frequently isolated pathogens were methicillin-susceptible Staphylococcus aureus (MSSA, n=18), fungal species (Fungi, n=8), methicillin-resistant Staphylococcus aureus (MRSA, n=5), and mixed infections (n=5). Stratified by joint type, among 67 knee PJI cases, 37 (55.2%) were culture-positive, with MSSA (n=13), fungi (n=6), and MRSA (n=3) being the most common pathogens. Among 30 hip PJI cases, 18 (60%) were culture-positive, with MSSA (n=5) and MRSA (n=1) as the predominant pathogens, while other bacterial species were distributed relatively evenly. Streptococcus spp., Enterococcus spp., methicillin-susceptible Staphylococcus epidermidis (MSSE), and other staphylococcal species were also detected in both joint types.

### 3.3 Laboratory test results

**Table 2** presents a comparison of SF-AGR, SE-AGR, ESR, CRP levels, and SF-PMN% between the aseptic revision group and the PJI group. The results show that ESR and CRP levels in the PJI group were significantly higher than those in the aseptic revision group. The median ESR in the PJI group was 59 (IQR 28-79), which was higher than the 13 (IQR 6-32) in the aseptic group; the median CRP in the PJI group was 44.9 (IQR 15.3-80), which was higher than the 6.94 (IQR 3.97-15) in the aseptic group. These differences were statistically significant (P < 0.0001). The SF-PMN% in the PJI group was significantly higher, with a median of 88 (IQR 83-92), compared to 50 (IQR 26-72) in the aseptic group (P < 0.0001). The SF-AGR and SE-AGR in the PJI group were significantly lower than those in the aseptic revision group. The median SF-AGR in the PJI group was 1.3 (IQR 1-1.5), compared to 2.1 (IQR 1.7-2.5) in the aseptic group (P < 0.0001). The median SE-AGR in the PJI group was 1.3 (IQR 1.1-1.4), which was significantly higher than the 1.7 (IQR 1.5-1.9) in the aseptic group (P < 0.0001).

**Table 2:** Characteristics of patients with or without periprosthetic joint infection.

Biomarkers	AF(n=133)	PJI(n=97)	P value
SF-AGR	2.1(IQR 1.7-2.5)	1.3(IQR 1-1.5)	<0.0001
SE-AGR	1.7(IQR 1.5-1.9)	1.3(IQR 1.1-1.4)	<0.0001
ESR (mm/h)	13(IQR 6-32)	59(IQR 28-79)	<0.0001
CRP(mg/L)	6.94(IQR 3.97-15)	44.9(IQR 15.3-80)	<0.0001
SF-PMN%	50(IQR 26-72)	88(IQR 83-92)	<0.0001

SF-AGR, albumin-to-globulin ratio in synovial fluid; SE-AGR, Serum Albumin-to-Globulin Ratio; CRP, C-reactive protein; ESR, erythrocyte sedimentation rate; SF-PMN%, polymorphonuclear cell percentage in synovial fluid; IQR  interquartile range.

### 3.4 Biomarker logistic regression analysis

Univariate logistic regression analysis demonstrated that all candidate biomarkers were significantly associated with PJI (all p < 0.0001) (**Table 3**). Because biomarker distributions were markedly skewed, all variables were log-transformed using the natural logarithm (LN) function prior to analysis to improve model stability and reliability. Among them, SF-AGR showed a strong negative correlation with PJI (β = −11.770, Wald χ^2^ = 47.3, OR = 7.758e-006), indicating that each unit increase in SF-AGR was associated with a markedly decreased odds of developing PJI. Similarly, the SE-AGR was also negatively correlated with infection. In contrast, ESR, CRP, and SF-PMN% were positively correlated with PJI, suggesting that lower SF-AGR and SE-AGR levels, together with higher ESR, CRP, and SF-PMN% values, indicate an increased risk of infection. In terms of effect strength (Wald χ^2^), SF-AGR exhibited a slightly lower value than SE-AGR (52.7), ESR (51.2), and CRP (60.7), but higher than SF-PMN% (36.8). Nevertheless, its effect remained highly significant (p < 0.0001), confirming a strong association between SF-AGR and PJI occurrence.

**Table 3:** Logistic regression analysis of inflammatory markers.

Biomarkers	β	SE	Wald χ^2^	P Value	OR	95% *CI*
SF-AGR	-11.770	1.712	47.3	<0.0001	7.758e-006	1.757e-007 to 0.0001527
SE-AGR	-8.009	1.103	52.7	<0.0001	0.0003323	3.197e-005 to 0.002452
ESR	1.624	0.227	51.2	<0.0001	5.075	3.349 to 8.182
CRP	1.341	0.172	60.7	<0.0001	3.821	2.781 to 5.467
SF-PMN%	5.306	0.875	36.8	<0.0001	201.5	42.63 to 1327

SE, standard error; OR, odds ratio; β, regression coefficient; 95% CI, 95% confidence interval. PJI, periprosthetic joint infection; SF-AGR, albumin-to-globulin ratio in synovial fluid; SE-AGR, Serum Albumin-to-Globulin Ratio; CRP, C-reactive protein; ESR, erythrocyte sedimentation rate. SF-PMN%, polymorphonuclear cell percentage in synovial fluid.

### 3.5 Receiver operating characteristic curve

**Figure 3:**
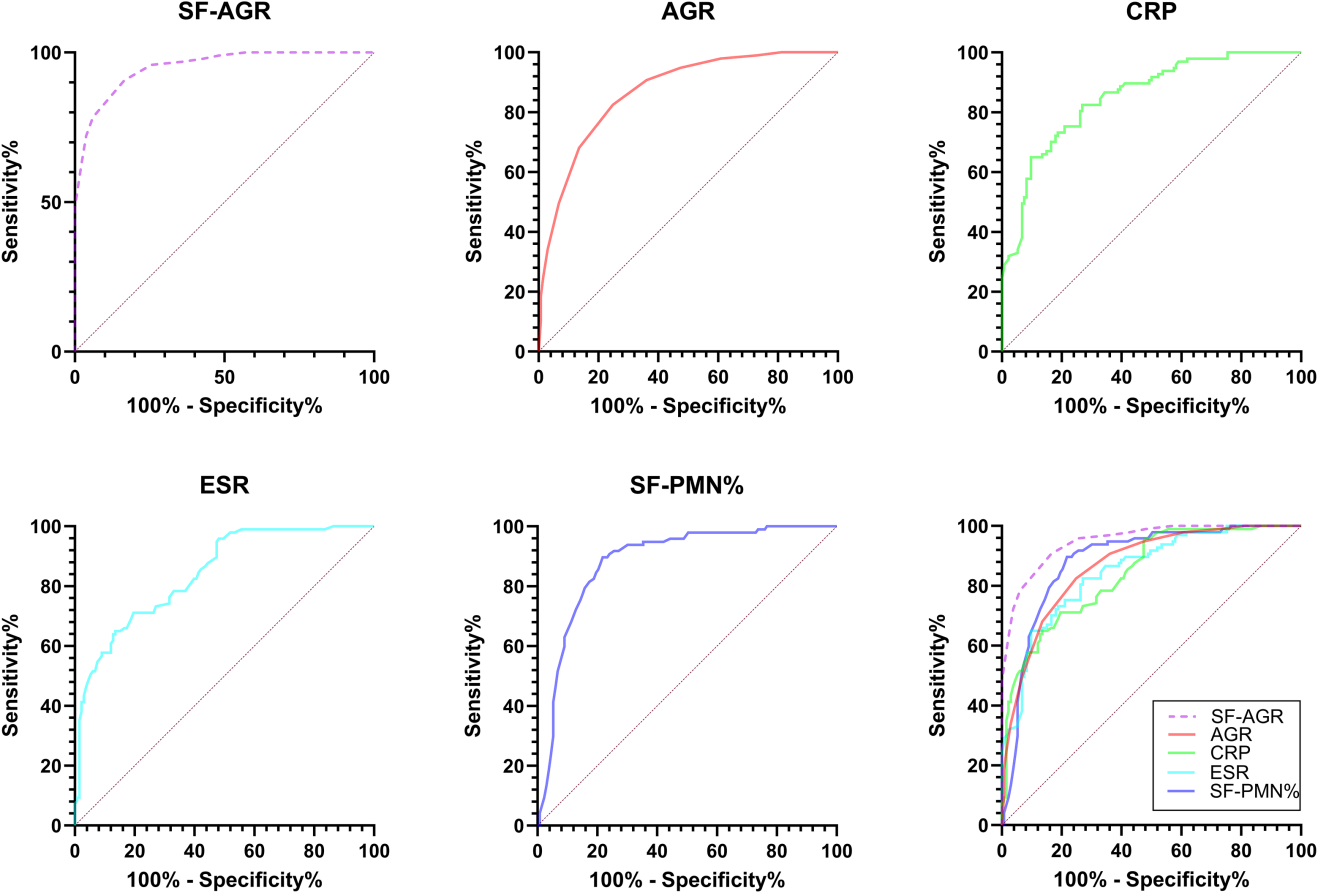
The receiver operating characteristic (ROC) curves show the PJI predictive value of SF-AGR, SE-AGR, CRP, ESR and SF-PMN%. PJI, periprosthetic joint infection; SF-AGR, albumin-to-globulin ratio in synovial fluid; SE-AGR, Serum Albumin-to-Globulin Ratio; CRP, C-reactive protein; ESR, erythrocyte sedimentation rate. SF-PMN%, polymorphonuclear cell percentage in synovial fluid.

We used the PJI group as the positive sample and the aseptic revision group as the negative sample, and then performed feature modeling based on SF-AGR, SE-AGR, ESR, CRP, and SF-PMN% (**Figure 3**). ROC curve analysis revealed that the AUC for SF-AGR, SE-AGR, ESR, CRP, and SF-PMN% were 0.9504, 0.8682, 0.8427, 0.8507, and 0.8843, respectively. Among these, SF-AGR had the highest AUC, demonstrating the best diagnostic performance.

**Table 4** presents the performance parameters of five diagnostic methods for diagnosing PJI. According to ROC curve analysis, SF-AGR performed the best in diagnosing PJI, with an AUC of 0.9504 and an optimal cutoff value of 1.65, demonstrating the highest diagnostic accuracy. The sensitivity of SF-AGR was 90.72%, specificity 83.46%, positive predictive value (PPV) 80.00%, negative predictive value (NPV) 92.5%, and accuracy 86.52%. SE-AGR had an AUC of 0.8682 and an optimal cutoff value of 1.45, with sensitivity 82.47%, specificity 75.19%, PPV 70.80%, NPV 85.47%, and accuracy 78.26%. The AUC for ESR was 0.8427, with an optimal cutoff value of 35.5, sensitivity 71.13%, specificity 80.45%, PPV 72.63%, NPV 79.26%, and accuracy 76.52%. The AUC for CRP was 0.8507, with an optimal cutoff value of 25.25, sensitivity 64.95%, specificity 90.30%, PPV 83.00%, NPV 77.94%, and accuracy 79.61%. The AUC for PMN% was 0.8843, with an optimal cutoff value of 74.5, sensitivity 89.69%, specificity 78.20%, PPV 75.00%, NPV 91.23%, and accuracy 83.05%. The diagnostic accuracy of SF-AGR for PJI was higher than that of SE-AGR, ESR, CRP, and SF-PMN%.

**Table 4:** Performance parameters of the five diagnostic methods for PJI.

Parameters	AUC(95% CI)	Best threshold	Sensitivity (95% CI)	Specificity (95% CI)	PPV (%)	NPV (%)	Accuracy (%)
SF-AGR	0.9504(0.9255-0.9753)	1.65	90.72(83.30-95.04)	83.46(76.22-88.82)	80.00	92.5	86.52
SE-AGR	0.8682(0.8228-0.9136)	1.45	82.47(73.71-88.76)	75.19(67.21-81.75)	70.80	85.47	78.26
ESR	0.8427(0.7931-0.8923)	35.5	71.13(61.45-79.21)	80.45(72.90-86.30)	72.63	79.26	76.52
CRP	0.8507(0.8020-0.8994)	25.25	64.95(55.05-73.71)	90.30(84.11-94.24)	83.00	77.94	79.61
SF-PMN%	0.8843(0.8395-0.9290)	74.5	89.69(82.05-94.30)	78.20(70.44-84.37)	75.00	91.23	83.05

SF-AGR, albumin-to-globulin ratio in synovial fluid; SE-AGR, Serum Albumin-to-Globulin Ratio; CRP, C-reactive protein; ESR, erythrocyte sedimentation rate; SF-PMN%, polymorphonuclear cell percentage in synovial fluid; IQR interquartile range; IC, iodine concentration; CI, confidence interval; PPV, positive predictive value; NPV, negative predictive value.

### 3.6 Subgroup analysis by joint type

To evaluate whether the diagnostic performance of biomarkers differed by joint type, ROC curve analyses were conducted separately for knee and hip PJI cases. Among 67 knee PJI cases (**Table 5**), SF-AGR showed the highest diagnostic performance, with an AUC of 0.9583, optimal cutoff of 1.65, sensitivity of 91.0%, specificity of 89.0%, positive predictive value (PPV) of 87.1%, negative predictive value (NPV) of 92.4%, and overall accuracy of 89.9%. SE-AGR, ESR, CRP, and SF-PMN% had slightly lower performance, with AUCs of 0.8689, 0.8578, 0.8507, and 0.8992, respectively. Among 30 hip PJI cases (**Table 6**), SF-AGR also had the highest AUC (0.9412) with an optimal cutoff of 1.55, sensitivity of 80.0%, specificity of 92.2%, PPV of 85.7%, NPV of 88.7%, and accuracy of 87.7%. SE-AGR, ESR, CRP, and SF-PMN% demonstrated AUCs of 0.8682, 0.8082, 0.8402, and 0.8480, respectively. Overall, although the optimal cutoffs and performance metrics of the biomarkers showed slight differences between TKA and THA, SF-AGR consistently demonstrated superior diagnostic performance for PJI in both joint types. This indicates that SF-AGR is a reliable synovial biomarker for distinguishing PJI from aseptic failure across different joints.

**Table 5:** Performance parameters of the five diagnostic methods for knee PJI.

Parameters(Knee)	AUC(95% CI)	Best threshold	Sensitivity (95% CI)	Specificity (95% CI)	PPV (%)	NPV (%)	Accuracy (%)
SF-AGR	0.9583(0.9299-0.9868)	1.65	91.04(81.81-95.83)	89.02(80.44-94.12)	87.14	92.40	89.93
SE-AGR	0.8689(0.8123-0.9254)	1.35	70.15(58.34-79.77)	86.59(77.55-92.34)	81.04	78.02	79.20
ESR	0.8578(0.8003-0.9152)	42.5	68.66(56.80-78.49)	86.59(77.55-92.34)	80.71	77.18	78.53
CRP	0.8507(0.8020-0.8994)	26.85	68.66(56.80-78.49)	87.80(78.99-93.24)	82.14	77.42	79.19
SF-PMN%	0.8992(0.8455-0.9529)	74	76.83(66.62-84.63)	95.52(87.64-98.78)	93.34	83.46	87.12

**Table 6:** Performance parameters of the five diagnostic methods for hip PJI.

Parameters(Hip)	AUC(95% CI)	Best threshold	Sensitivity (95% CI)	Specificity (95% CI)	PPV (%)	NPV (%)	Accuracy (%)
SF-AGR	0.9412(0.8939-0.9885)	1.55	80(62.69-90.49)	92.16(81.50-96.91)	85.72	88.68	87.66
SE-AGR	0.8682(0.8228-0.9136)	1.45	83.33(66.44-92.66)	78.43(65.37-87.51)	69.44	88.89	80.24
ESR	0.8082(0.7105-0.9058)	35.5	66.67(48.78-80.77)	82.35(69.75-90.43)	68.96	80.77	76.54
CRP	0.8402(0.7508-0.9296)	10.6	80(62.69-90.49)	78.43(65.37-87.51)	68.57	86.96	79.01
SF-PMN%	0.8480(0.7596-0.9365)	75.5	76.67(59.07-88.21)	80.39(67.54-88.98)	69.70	85.42	79.01

## 4 Discussion

PJI is one of the most severe complications following joint replacement surgery (Tande and Patel, 2014). Despite advancements in diagnostic criteria and technologies, early diagnosis of PJI remains a significant challenge. Immune evasion mechanisms can cause clinical manifestations of infection that resemble those of aseptic loosening, leading to delayed PJI diagnosis (Akcaalan et al., 2022). Although traditional serological markers such as CRP and ESR are commonly used in PJI diagnosis, their effectiveness is often limited in cases of chronic infections, culture-negative pathogens, or PJI caused by immune system abnormalities, which compromises both sensitivity and specificity (Malekzadeh et al., 2010; Pérez-Prieto et al., 2017; Parvizi et al., 2018; Watanabe et al., 2021). This study evaluated the diagnostic value of albumin-to-globulin ratio (AGR) in both synovial fluid and serum for chronic PJI and found that SF-AGR demonstrated a significant advantage in improving diagnostic accuracy.

Univariate logistic regression analysis demonstrated that all five candidate biomarkers were significantly associated with PJI (all p < 0.0001). Among them, SF-AGR showed a strong inverse association with infection, indicating that lower SF-AGR levels were closely related to the presence of PJI. Despite slightly lower Wald χ^2^ values compared with some serum markers, SF-AGR remained highly significant, suggesting a robust association that may reflect complex local intra-articular dynamics. ROC curve analysis further confirmed that SF-AGR had the best overall diagnostic performance among all evaluated biomarkers, with superior sensitivity and overall accuracy compared with ESR, CRP, and SF-PMN%. In addition, SF-AGR outperformed serum AGR, highlighting the advantage of synovial-based markers in reflecting the local infectious environment. Subgroup analyses showed small differences between TKA and THA, but these did not change the overall diagnostic ranking of the biomarkers, with SF-AGR consistently performing best. Such variations are likely related to joint-specific factors, including differences in local inflammatory responses and synovial fluid characteristics. The consistent performance of SF-AGR across joint types supports its general applicability in the diagnosis of chronic PJI (Choe et al., 2023). Taken together, these findings indicate that SF-AGR is a reliable and practical biomarker for distinguishing chronic PJI from aseptic failure. Given its accessibility, low cost, and compatibility with routine laboratory testing, SF-AGR may serve as a promising tool for preoperative diagnosis, including in resource-limited settings.

In PJI, a substantial body of literature has demonstrated that serum AGR possesses certaindiagnostic value. A meta-analysis indicated that serum AGR has a certain diagnostic effectiveness indiagnosing PJI, but its sensitivity and specificity were only 75.3% and 75.7%, respectively (Choe et al., 2023). Similarly, in Domenico’s study, although serum AGR showed a higher sensitivity (95%) at an optimal cutoff value of 1.43, its specificity was only 63% (De Mauro et al., 2025). In another retrospective study, SE-AGR demonstrated a specificity of 86.7% but a sensitivity of only 67.1% for diagnosing PJI (Shang et al., 2022). The variable diagnostic performance of SE-AGR reported in different studies may be influenced by acute or chronic inflammation related to other organs or systems, as well as by antibiotic treatment, systemic infections, trauma, or surgical interventions, which can limit its utility in PJI diagnosis (Tripathi et al., 2023; Hausfater, 2014). Therefore, the diagnostic accuracy of SE-AGR for PJI requires further validation.

In this study, we report for the first time that SF-AGR outperforms serum AGR across all diagnostic parameters for PJI. This superiority may be explained by several factors. On one hand, immune cells in the joint, such as macrophages, neutrophils, and synovial cells, are activated during infection and release a large number of inflammatory mediators, immunoglobulins, and chemokines (Boff et al., 2018; Bai et al., 2022). These substances accumulate in the synovial fluid, directly reflecting the inflammatory activity at the site of the lesion. In contrast, serum levels are often diluted due to systemic circulation and masked by liver and kidney metabolism (James et al., 2022; Hall, 2025; Kluzek and O’Sullivan, 2025). Synovial fluid, as a direct specimen from the joint cavity, can more accurately reflect local immune and inflammatory responses, making its diagnostic value superior to that of serum biomarkers (Zhou et al., 2025). On the other hand, the joint cavity is a relatively closed anatomical space, with the protein concentration in synovial fluid being about one-third of that in plasma (Hui et al., 2012). Plasma components can only significantly enter the synovial fluid during inflammation or infection when vascular permeability is increased (Scanzello and Goldring, 2012; Solarino et al., 2022). As a result, abnormalities detected in synovial fluid are more closely related to the local pathological state. This “barrier effect” makes synovial fluid biomarkers more specific than systemic serum markers. For example, in studies using serum and synovial fluid calprotectin as biomarkers for diagnosing chronic hip and knee periprosthetic joint infections, synovial fluid calprotectin demonstrated better sensitivity and specificity (Grzelecki et al., 2021). In another study, both serum CRP and synovial fluid CRP helped in diagnosing PJI, but synovial fluid CRP showed higher accuracy and better diagnostic performance compared to serum CRP (Li et al., 2024). Therefore, compared with serum biomarkers, synovial biomarkers obtained directly from the infected joint provide more reliable and accurate diagnostic information for PJI. Our findings further indicate that SF-AGR, relative to traditional markers such as ESR, CRP, SF-PMN%, and even potential SE-AGR, has superior clinical value in diagnosing PJI.

Although the results of this study indicate that SF-AGR has significant advantages in diagnosing PJI, there are several limitations to consider: (1) The sample size in this study was relatively small, and it was a single-center study. Future studies with larger sample sizes and multi-center designs are needed to further validate the reliability of these findings; (2) Although we excluded patients with inflammatory arthritis and those with concomitant infections, other potential confounders may still influence the performance of SF-AGR. These factors include the patient’s immune status, comorbidities such as diabetes and cardiovascular diseases, and medication history, including long-term use of immunosuppressive drugs and antibiotics. Future research should explore the impact of these factors on the diagnostic accuracy of SF-AGR; (3) In this study, PJI was diagnosed according to the 2013 MSIS criteria. While these criteria are widely accepted and practical in both clinical and research settings, they do not include some of the newer biomarkers and diagnostic parameters. Consequently, the potential relationship between SF-AGR and these additional markers was not explored in our study; (4) This study focused exclusively on chronic PJI, and the potential utility of SF-AGR in acute PJI was not evaluated. The applicability of SF-AGR across different stages of PJI warrants further investigation; (5) Our cohort had a high proportion of Staphylococcus aureus, a high-virulence pathogen. Therefore, these findings may not be generalizable to populations dominated by low-virulence organisms; (6) This study focused solely on the role of SF-AGR as a single biomarker. However, in clinical practice, diagnosing infections often requires a comprehensive evaluation of multiple biomarkers and clinical symptoms. Future studies could further investigate the combined use of SF-AGR with other biomarkers, such as IL-6, CRP, ESR, and white blood cell count, to effectively improve diagnostic sensitivity and specificity.

## 5 Conclusion

This study demonstrates that AGR in synovial fluid from the knee or hip joint has significant clinical value for the diagnosis of PJI. SF-AGR, as a novel synovial biomarker, shows reliable diagnostic performance in distinguishing PJI from aseptic failure. These findings highlight its potential utility as an adjunct to existing diagnostic methods. Additional studies are needed to confirm these findings and further evaluate the potential clinical applications of SF-AGR.

## Data Availability

The original contributions presented in the study are included in the article/supplementary material. Further inquiries can be directed to the corresponding authors.

## References

[B1] Akcaalan S. Ozaslan H. I. Caglar C. Şimşek M. E. Citak M. Akkaya M. (2022). Role of biomarkers in periprosthetic joint infections. Diagn Basel Switz 12, 2958. doi: 10.3390/diagnostics12122958. PMID: 36552965 PMC9777153

[B2] Bai L.-K. Su Y.-Z. Wang X.-X. Bai B. Zhang C.-Q. Zhang L.-Y. . (2022). Synovial macrophages: past life, current situation, and application in inflammatory arthritis. Front. Immunol. 13. doi: 10.3389/fimmu.2022.905356. PMID: 35958604 PMC9361854

[B3] Boff D. Crijns H. Teixeira M. M. Amaral F. A. Proost P. (2018). Neutrophils: beneficial and harmful cells in septic arthritis. Int. J. Mol. Sci. 19, 468. doi: 10.3390/ijms19020468. PMID: 29401737 PMC5855690

[B4] Boyle K. K. Kapadia M. Landy D. C. Henry M. W. Miller A. O. Westrich G. H. (2020). Utilization of debridement, antibiotics, and implant retention for infection after total joint arthroplasty over a decade in the United States. J. Arthroplasty 35, 2210–2216. doi: 10.1016/j.arth.2020.03.029. PMID: 32279946

[B5] Boyle K. K. Wood S. Tarity T. D. (2018). Low-virulence organisms and periprosthetic joint infection-biofilm considerations of these organisms. Curr. Rev. Musculoskelet. Med. 11, 409–419. doi: 10.1007/s12178-018-9503-2. PMID: 29961193 PMC6105490

[B6] Choe H. Kamono E. Abe K. Hieda Y. Ike H. Kumagai K. . (2023). Accuracy of albumin, globulin, and albumin–globulin ratio for diagnosing periprosthetic joint infection: a systematic review and meta-analysis. J. Clin. Med. 12, 7512. doi: 10.3390/jcm12247512. PMID: 38137581 PMC10743640

[B7] Costerton J. W. Stewart P. S. Greenberg E. P. (1999). Bacterial biofilms: a common cause of persistent infections. Science 284, 1318–1322. doi: 10.1126/science.284.5418.1318. PMID: 10334980

[B8] De Mauro D. Ascione T. Festa E. Marasco L. Leggieri F. Rosito S. . (2025). Diagnostic work-up in periprosthetic joint infections of the knee: can the albumin-to-globulin ratio be a screening tool? J. Orthop. Traumatol Off. J. Ital Soc Orthop. Traumatol 26, 39. doi: 10.1186/s10195-025-00857-8. PMID: 40603631 PMC12222592

[B9] Dong M. Wang Y. Fan H. Yang D. Wang R. Feng Y. (2024). The albumin to globulin ratio performs well for diagnosing periprosthetic joint infection: a single-center retrospective study. J. Arthroplasty 39, 229–235.e4. doi: 10.1016/j.arth.2023.08.002. PMID: 37557968

[B10] Doweiko J. P. Nompleggi D. J. (1991). Role of albumin in human physiology and pathophysiology. JPEN J. Parenter Enteral Nutr. 15, 207–211. doi: 10.1177/0148607191015002207. PMID: 2051560

[B11] Gabay C. Kushner I. (1999). Acute-phase proteins and other systemic responses to inflammation. N. Engl. J. Med. 340, 448–454. doi: 10.1056/NEJM199902113400607. PMID: 9971870

[B12] Grzelecki D. Walczak P. Szostek M. Grajek A. Rak S. Kowalczewski J. (2021). Blood and synovial fluid calprotectin as biomarkers to diagnose chronic hip and knee periprosthetic joint infections. Bone Jt J. 103-B, 46–55. doi: 10.1302/0301-620X.103B1.BJJ-2020-0953.R1. PMID: 33380202

[B13] Gu Q. Gu A. Zhang J. Zhou Y. Shen C. (2025). Medical imaging diagnosis of orthopedic prosthesis-associated infections: a narrative review. Quant Imaging Med. Surg. 15, 947–961. doi: 10.21037/qims-24-403. PMID: 39839020 PMC11744130

[B14] Hall A. M. (2025). Protein handling in kidney tubules. Nat. Rev. Nephrol 21, 241–252. doi: 10.1038/s41581-024-00914-1. PMID: 39762367

[B15] Hallab N. J. Jacobs J. J. (2009). Biologic effects of implant debris. Bull. NYU Hosp Jt Dis. 67, 182–188. doi: 10.1016/j.esas.2009.11.005. PMID: 19583551

[B16] Hausfater P. (2014). Biomarkers and infection in the emergency unit. Med. Mal Infect. 44, 139–145. doi: 10.1016/j.medmal.2014.01.002. PMID: 24556451

[B17] Hoveidaei A. Tavakoli Y. Ramezanpour M. R. Omouri-kharashtomi M. Taghavi S. P. Hoveidaei A. H. . (2024). Imaging in periprosthetic joint infection diagnosis: a comprehensive review. Microorganisms 13, 10. doi: 10.3390/microorganisms13010010. PMID: 39858778 PMC11768089

[B18] Hui A. Y. McCarty W. J. Masuda K. Firestein G. S. Sah R. L. (2012). A systems biology approach to synovial joint lubrication in health, injury, and disease. Wiley Interdiscip Rev. Syst. Biol. Med. 4, 15–37. doi: 10.1002/wsbm.157. PMID: 21826801 PMC3593048

[B19] James B. H. Papakyriacou P. Gardener M. J. Gliddon L. Weston C. J. Lalor P. F. (2022). The contribution of liver sinusoidal endothelial cells to clearance of therapeutic antibody. Front. Physiol. 12. doi: 10.3389/fphys.2021.753833. PMID: 35095549 PMC8795706

[B20] Kluzek S. O’Sullivan O. (2025). Novel method for routine ultrasound-guided serum collection for biomarker analysis around the knee joint. F1000Research 14, 68. doi: 10.12688/f1000research.159920.1. PMID: 40893738 PMC12398681

[B21] Kurtz S. M. Lau E. Watson H. Schmier J. K. Parvizi J. (2012). Economic burden of periprosthetic joint infection in the United States. J. Arthroplasty 27, 61–65.e1. doi: 10.1016/j.arth.2012.02.022. PMID: 22554729

[B22] Li N. Kagan R. Hanrahan C. J. Hansford B. G. (2020). Radiographic evidence of soft-tissue gas 14 days after total knee arthroplasty is predictive of early prosthetic joint infection. AJR Am. J. Roentgenol 214, 171–176. doi: 10.2214/AJR.19.21702. PMID: 31573855

[B23] Li F. Zhou H. Yang Y. Yang J. Wang H. Hu N. (2024). Diagnostic and predictive efficacy of synovial fluid versus serum C-reactive protein levels for periprosthetic joint infection and reimplantation success. J. Arthroplasty 39, 1932–1938. doi: 10.1016/j.arth.2024.04.054. PMID: 38670172

[B24] Malekzadeh D. Osmon D. R. Lahr B. D. Hanssen A. D. Berbari E. F. (2010). Prior use of antimicrobial therapy is a risk factor for culture-negative prosthetic joint infection. Clin. Orthop. 468, 2039–2045. doi: 10.1007/s11999-010-1338-0. PMID: 20401555 PMC2895855

[B25] Marang-van de Mheen P. J. Bragan Turner E. Liew S. Mutalima N. Tran T. Rasmussen S. . (2017). Variation in prosthetic joint infection and treatment strategies during 4.5 years of follow-up after primary joint arthroplasty using administrative data of 41397 patients across Australian, European and United States hospitals. BMC Musculoskelet. Disord. 18, 207. doi: 10.1186/s12891-017-1569-2. PMID: 28532409 PMC5441102

[B26] Mitsui H. Choe H. Shimoda M. Yamane H. Hieda Y. Abe K. . (2025). Evaluation of the diagnostic accuracy of serum albumin and globulin in pyogenic spondylitis. J. Clin. Med. 14, 6001. doi: 10.3390/jcm14176001. PMID: 40943760 PMC12429411

[B27] Palan J. Nolan C. Sarantos K. Westerman R. King R. Foguet P. (2019). Culture-negative periprosthetic joint infections. EFORT Open Rev. 4, 585–594. doi: 10.1302/2058-5241.4.180067. PMID: 31754464 PMC6836077

[B28] Parvizi J. Tan T. L. Goswami K. Higuera C. Della Valle C. Chen A. F. . (2018). The 2018 definition of periprosthetic hip and knee infection: an evidence-based and validated criteria. J. Arthroplasty 33, 1309–1314.e2. doi: 10.1016/j.arth.2018.02.078. PMID: 29551303

[B29] Pérez-Prieto D. Portillo M. E. Puig-Verdié L. Alier A. Martínez S. Sorlí L. . (2017). C-reactive protein may misdiagnose prosthetic joint infections, particularly chronic and low-grade infections. Int. Orthop. 41, 1315–1319. doi: 10.1007/s00264-017-3430-5. PMID: 28321490

[B30] Qin L. Li X. Wang J. Gong X. Hu N. Huang W. (2020). Improved diagnosis of chronic hip and knee prosthetic joint infection using combined serum and synovial IL-6 tests. Bone Jt Res. 9, 587–592. doi: 10.1302/2046-3758.99.BJR-2020-0095.R1. PMID: 33005398 PMC7502257

[B31] Qin L. Yang S. Zhao C. Yang J. Li F. Xu Z. . (2024). Prospects and challenges for the application of tissue engineering technologies in the treatment of bone infections. Bone Res. 12, 28. doi: 10.1038/s41413-024-00332-w. PMID: 38744863 PMC11094017

[B32] Scanzello C. R. Goldring S. R. (2012). The role of synovitis in osteoarthritis pathogenesis. Bone 51, 249–257. doi: 10.1016/j.bone.2012.02.012. PMID: 22387238 PMC3372675

[B33] Shang G. Fei Z. Xu H. Wang Y. Xiang S. (2022). Globulin and albumin to globulin ratio precisely diagnose periprosthetic joint infection and determine the timing of second-stage reimplantation. J. Orthop. Surg. 17, 12. doi: 10.1186/s13018-021-02899-0. PMID: 34991649 PMC8740003

[B34] Solarino G. Bizzoca D. Moretti L. Vicenti G. Piazzolla A. Moretti B. (2022). What’s new in the diagnosis of periprosthetic joint infections: focus on synovial fluid biomarkers. Trop. Med. Infect. Dis. 7, 355. doi: 10.3390/tropicalmed7110355. PMID: 36355897 PMC9692966

[B35] Su X. Chen Y. Zhan Q. Zhu B. Chen L. Zhao C. . (2022). The ratio of IL-6 to IL-4 in synovial fluid of knee or hip performances a noteworthy diagnostic value in prosthetic joint infection. J. Clin. Med. 11, 6520. doi: 10.3390/jcm11216520. PMID: 36362748 PMC9654466

[B36] Tande A. J. Patel R. (2014). Prosthetic joint infection. Clin. Microbiol. Rev. 27, 302–345. doi: 10.1128/CMR.00111-13. PMID: 24696437 PMC3993098

[B37] Tripathi S. Tarabichi S. Parvizi J. Rajgopal A. (2023). Current relevance of biomarkers in diagnosis of periprosthetic joint infection: an update. Arthroplasty. 5, 41. doi: 10.1186/s42836-023-00192-5 37525262 PMC10391917

[B38] Uhel F. Corvaisier G. Poinsignon Y. Chirouze C. Beraud G. Grossi O. . (2019). Mycobacterium tuberculosis prosthetic joint infections: a case series and literature review. J. Infect. 78, 27–34. doi: 10.1016/j.jinf.2018.08.008. PMID: 30138639

[B39] Villa J. M. Pannu T. S. Piuzzi N. Riesgo A. M. Higuera C. A. (2020). Evolution of diagnostic definitions for periprosthetic joint infection in total hip and knee arthroplasty. J. Arthroplasty 35, S9–S13. doi: 10.1016/j.arth.2019.10.032. PMID: 32046836

[B40] Wang Z. Mao H. Xu G. (2023). Fibrinogen, albumin-to-globulin ratio, and fibrinogen to albumin-to-globulin ratio may be potential diagnostic biomarkers for infected tibial nonunion. Int. Immunopharmacol 121, 110542. doi: 10.1016/j.intimp.2023.110542. PMID: 37356122

[B41] Wang H. Zhou H. Jiang R. Qian Z. Wang F. Cao L. (2021). Globulin, the albumin-to-globulin ratio, and fibrinogen perform well in the diagnosis of periprosthetic joint infection. BMC Musculoskelet. Disord. 22, 583. doi: 10.1186/s12891-021-04463-7. PMID: 34172035 PMC8235840

[B42] Watanabe S. Kobayashi N. Tomoyama A. Choe H. Yamazaki E. Inaba Y. (2021). Clinical characteristics and risk factors for culture-negative periprosthetic joint infections. J. Orthop. Surg. Res. 16, 292. doi: 10.1186/s13018-021-02450-1. PMID: 33941220 PMC8091510

[B43] Zhang J. Wang T. Fang Y. Wang M. Liu W. Zhao J. . (2021). Clinical significance of serum albumin/globulin ratio in patients with pyogenic liver abscess. Front. Surg. 8. doi: 10.3389/fsurg.2021.677799. PMID: 34917645 PMC8669143

[B44] Zhou H. Hu Q. Zhang R. Yang Y. Li F. Yang J. . (2025). Evaluation of heparin-binding protein as a novel biomarker for the detection of periprosthetic joint infection. Front. Cell. Infect. Microbiol. 15. doi: 10.3389/fcimb.2025.1651759. PMID: 41459156 PMC12738923

[B45] Zmistowski B. Karam J. A. Durinka J. B. Casper D. S. Parvizi J. (2013). Periprosthetic joint infection increases the risk of one-year mortality. J. Bone Joint Surg. Am. 95, 2177–2184. doi: 10.2106/JBJS.L.00789. PMID: 24352771

